# Effects of dietary *Bacillus amyloliquefaciens* supplementation on growth performance, intestinal morphology, inflammatory response, and microbiota of intra-uterine growth retarded weanling piglets

**DOI:** 10.1186/s40104-018-0236-2

**Published:** 2018-03-13

**Authors:** Yue Li, Hao Zhang, Weipeng Su, Zhixiong Ying, Yueping Chen, Lili Zhang, Zhaoxin Lu, Tian Wang

**Affiliations:** 1 College of Animal Science and Technology, Nanjing Agricultural University, Nanjing, Jiangsu 210095 China; 2 College of Food Science and Technology, Nanjing Agricultural University, Nanjing, Jiangsu 210095 China

**Keywords:** Apoptosis, *Bacillus amyloliquefaciens*, Immune status, Intra-uterine growth retardation, Microbiota, Piglet, Small intestine

## Abstract

**Background:**

The focus of recent research has been directed toward the probiotic potential of *Bacillus amyloliquefaciens* (BA) on the gut health of animals. However, little is known about BA’s effects on piglets with intra-uterine growth retardation (IUGR). Therefore, this study investigated the effects of BA supplementation on the growth performance, intestinal morphology, inflammatory response, and microbiota of IUGR piglets.

**Methods:**

Eighteen litters of newborn piglets were selected at birth, with one normal birth weight (NBW) and two IUGR piglets in each litter (i.e., 18 NBW and 36 IUGR piglets in total). At weaning, the NBW piglet and one of the IUGR piglets were assigned to groups fed a control diet (i.e., the NBW-CON and IUGR-CON groups). The other IUGR piglet was assigned to a group fed the control diet supplemented with 2.0 g BA per kg of diet (i.e., IUGR-BA group). The piglets were thus distributed across three groups for a four-week period.

**Results:**

IUGR reduced the growth performance of the IUGR-CON piglets compared with the NBW-CON piglets. It was also associated with decreased villus sizes, increased apoptosis rates, reduced goblet cell numbers, and an imbalance between pro- and anti-inflammatory cytokines in the small intestine. Supplementation with BA improved the average daily weight gain and the feed efficiency of the IUGR-BA group compared with the IUGR-CON group (*P* < 0.05). The IUGR-BA group exhibited increases in the ratio of jejunal villus height to crypt depth, in ileal villus height, and in ileal goblet cell density. They also exhibited decreases in the numbers of jejunal and ileal apoptotic cells and ileal proliferative cells (*P* < 0.05). Supplementation with BA increased interleukin 10 content, but it decreased tumor necrosis factor alpha level in the small intestines of the IUGR-BA piglets (*P* < 0.05). Furthermore, compared with the IUGR-CON piglets, the IUGR-BA piglets had less *Escherichia coli* in their jejunal digesta, but more *Lactobacillus* and *Bifidobacterium* in their ileal digesta (*P* < 0.05).

**Conclusions:**

Dietary supplementation with BA improves morphology, decreases inflammatory response, and regulates microbiota in the small intestines of IUGR piglets, which may contribute to improved growth performance during early life.

**Electronic supplementary material:**

The online version of this article (10.1186/s40104-018-0236-2) contains supplementary material, which is available to authorized users.

## Background

Intra-uterine growth retardation (IUGR) is defined as impaired growth and development of the embryo/fetus or its organs during gestation [1]. Approximately 15–20% of neonatal piglets suffer from IUGR, which has been a significant problem in commercial swine production [2]. Neonates that have experienced IUGR are usually at increased risk of intestinal diseases, and this may be an underlying factor contributing to compromised growth performance and to higher mortality and morbidity during the neonatal period [3–5]. Although the exact etiology of neonatal intestinal diseases remains unclear, prematurity and abnormal bacterial colonization of the gastrointestinal tract, in parallel with loss of commensal microbiota are considered to be risk factors. In general, postnatal colonization of the piglet’s intestine begins with the appearance of *Escherichia coli*, *Lactobacilli*, and *Streptococci* [6]. The numbers of those organisms increase rapidly during the first few days, after which *Lactobacilli* become the predominant group, followed by *Enterobacteria*, *Streptococci*, *Bacteroides*, and *Clostridia*, in that order [7]. With the introduction of solid feed, anaerobes increase in number and diversity until a stable pattern is established. *Lactobacilli* remain a predominant part of the bacterial community, after a transient decrease during the weaning transition [8–11]. However, epidemiological and animal studies have indicated that IUGR results in a delay in the establishment of the normal gut bacterial community, with lower numbers of anaerobes, in general, and of *Lactobacilli* and *Bifidobacterium*, in particular. This may disturb the colonization resistance of commensal microbiota [12–15]. Moreover, perturbations in the normal pattern of implantation of microbiota may have adverse consequences on digestive and absorptive functions, as well as on the intestinal epithelial barrier [16–18]. This, in turn, may render mucosa susceptible to invasion by luminal bacteria, resulting in the disruption of defense mechanisms, and subsequently leading to intestinal disorders [19].

Numerous reports have suggested that probiotics are beneficial for maintaining intestinal homeostasis and host health [20–22]. In recent decades, several species of *Bacillus* have been found to promote growth, feed efficiency, and digestive function, and those species are becoming prevalent in swine and poultry feed [23–26]. Among them, *Bacillus amyloliquefaciens* (BA) has gained more attention, and emerging evidence has identified its health-beneficial properties in the treatment of intestinal disorders, such as diarrhea and inflammation. The likely mechanisms for this are immunomodulation, competitive exclusion of gastrointestinal pathogens, and secretion of the antimicrobial compounds that suppress the growth of harmful bacteria [27, 28]. However, to date, little evidence is available regarding the beneficial effects of BA on IUGR animals. Thus, the objective of the present research was to evaluate the effects of dietary supplementation with BA on the growth performance, intestinal morphology, immune status, and microbiota of IUGR weanling piglets. Pigs are well suited to acting as an animal model for IUGR clinical studies due to their biological similarities to humans, and this study may help in devising preventive strategies for neonatal intestinal diseases of IUGR offspring.

## Methods

### Experimental design, diets, and management

**Fig. 1:**
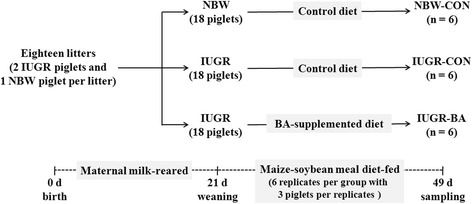
Schematic representation of the experimental procedures

All experiments were conducted in accordance with the guidelines established by the Institutional Animal Care and Use Committee of Nanjing Agricultural University (NJAU-CAST-2016-036). Eighty-two healthy sows with similar parity (second or third) and similar expected farrowing dates (the time between the expected earliest and latest farrowing dates was 4 d) were chosen for inclusion in the study. Eighteen litters of between 10 and 13 piglets were selected at birth, and two males in each litter met the selection criteria for IUGR. Newborn piglets [Duroc × (Landrace × Yorkshire)] with a birth weight (BW) close to the average BW of the herd [i.e., within 0.5 standard deviations (SD)] were identified as normal BW (NBW), and those with a 2 SD lower BW were defined as IUGR [16, 17, 29]. Two IUGR male piglets (0.91 ± 0.05 kg) and one NBW male piglet (1.50 ± 0.04 kg) were selected from each litter. All piglets were weaned at 21 d of age and transferred to the weaner unit. In each litter, the NBW piglet and one of the IUGR piglets were assigned to groups fed the control diet (the NBW-CON and the IUGR-CON group, respectively). The other IUGR piglet was assigned to the group fed the control diet supplemented with 2.0 g BA per kg of diet (the IUGR-BA group). The piglets remained in these three groups for a four-week period. Each group consisted of six replicates with three piglets per replicate, making a total of 18 piglets per group (Fig. 1). The strain of BA used in the current study was BA ES-2 (5.4 × 10^9^ colony-forming units per g), a wild-type strain originally isolated from the *Scutellaria* plant. Prior to this study, we carried out an independent experiment (unpublished) to determine the most appropriate of five possible doses (0.1 g, 0.2 g, 0.5 g, 1.0 g, or 2.0 g per kg of diet). We found that the optimum effect on the growth performance of NBW piglets was obtained when BA was provided at 1.0 g and 2.0 g per kg of diet. To ensure that the IUGR piglets would receive adequate amounts of BA, a dosage of 2.0 g per kg of diet was selected. This is because the feed intake of IUGR piglets is generally lower than that of NBW piglets during the first 4 wk after weaning. The ingredient composition and nutrient content of the diets (see Table 1) were formulated based on the NRC (2012) guidelines [30]. The piglets had free access to food and water during the feeding period. Their average daily weight gain (ADG) and average daily feed intake (ADFI) were recorded every week. Feed efficiency (FE) was calculated by dividing ADG by ADFI.

**Table 1:** Composition and nutrient level of the diet (as-fed basis)

Items^a^	Contents
Ingredients, %	
Maize	62.78
Soybean meal	15.00
Fermented soybean meal	7.00
Extruded soybean	7.00
Soy protein isolate	1.30
Soybean oil	2.00
CaHPO_4_	1.80
Limestone	0.80
Salt	0.35
L-lysine HCl, 78%	0.52
L-methionine	0.13
L-threonine	0.15
L-isoleucine	0.10
L-tryptophan	0.01
L-histidine	0.01
Calcium propionate, 50%	0.05
Premix^b^	1.00
Total	100.00
Calculated nutrient levels	
Digestible energy, Mcal/kg	3.47
Metabolizable energy, Mcal/kg	3.30
Crude protein	20.38
SID lysine	1.34
SID methionine	0.40
SID methionine + cystine	0.72
SID threonine	0.80
SID tryptophan	0.35
SID histidine	0.65
SID isoleucine	0.70
SID valine	1.04
Total calcium	0.82
Available phosphorus	0.43
Analyzed nutrient levels	
Crude protein	20.45
Total lysine	1.53
Total methionine	0.45
Total methionine + cystine	0.85
Total threonine	0.95
Total tryptophan	0.39
Total histidine	0.75
Total isoleucine	0.80
Total valine	1.20
Total calcium	0.81
Total phosphorus	0.66

^a^*SID* standard ileal digestible

^b^Provide the following per kg complete diet: Vitamin A, 8,000 IU; Vitamin D_3_, 3,000 IU; Vitamin E, 20 IU; Vitamin K_3_, 3 mg; Vitamin B_1_, 2 mg; Vitamin B_2_, 5 mg; Vitamin B_6_, 7 mg; Vitamin B_12_, 0.02 mg; Niacin, 30 mg; Pantothenic acid, 15 mg; Folic acid, 0.3 mg; Biotin, 0.08 mg; Choline chloride, 500 mg; Fe (from ferrous sulfate), 110 mg; Cu (from copper sulfate) 7 mg; Mn (from manganese sulfate), 5 mg; Zn (from zinc sulfate), 110 mg; I (from calcium iodate), 0.3 mg; Se (from sodium selenite), 0.3 mg

### Sample collection

At the end of the experiment, the piglet with body weight closest to the mean body weight of its replicate was selected (6 piglets per group, making 18 piglets in total). The selected piglets were killed by exsanguination after electrical stunning, following which their entire gastrointestinal tracts were rapidly removed. The small intestine was dissected free of the mesentery and placed on a chilled stainless steel tray. The proximal jejunum and the distal ileum were quickly excised. Their contents were collected, rapidly frozen in liquid nitrogen, and stored at − 80 °C until analysis. Segments measuring 3 cm were cut from the jejunum and the ileum and then flushed gently with ice-cold phosphate-buffered saline (PBS, pH = 7.4). They were then fixed in 4% fresh, chilled paraformaldehyde solution for histological measurements. Segments of the proximal jejunum and the distal ileum measuring approximately 20 cm were opened longitudinally, and their contents were flushed with ice-cold PBS. Mucosa was collected by scraping using a sterile glass microscope slide. The mucosa was then rapidly frozen in liquid nitrogen and stored at − 80 °C until analysis.

### Intestinal morphology analysis

After fixation for 24 h, the intestinal samples were dehydrated using a graded series of ethanol (70–100%) and cleared with xylene, and then the samples were embedded in paraffin. Cross sections of the segments were cut at a thickness of 5 μm and stained with hematoxylin and eosin. Villus height (VH) and crypt depth (CD) of fifteen well-oriented villi per segment were measured using a Nikon ECLIPSE 80i light microscope with a computer-assisted morphometric system (Nikon Corporation, Tokyo, Japan).

### Goblet cell staining

The samples for goblet cell staining were prepared in accordance with the procedures for the intestinal morphology analysis. The combined Alcian Blue/periodic acid Schiff stain technique was then employed to measure the intestinal goblet cell density [31]. In particular, deparaffinized and rehydrated sections were stained with 1.0% Alcian Blue solution (Alcian Blue in 3% acetic acid solution), gently washed in double-distilled H_2_O for 10 min, oxidized in 1.0% periodic acid solution for 15 min, rinsed again in double-distilled H_2_O for 10 min, and then placed in periodic acid Schiff solution for 30 min. Goblet cells were counted in fifteen well-oriented villi per section, using the Nikon ECLIPSE 80i light microscope (Nikon Corporation, Japan). Goblet cell density was calculated as the goblet cell count divided by the corresponding villus length, averaged and expressed as goblet cell number per 100 μm of villus length.

### Immunohistochemistry

Terminal deoxynucleotidyl transferase-mediated deoxyuridine triphosphate nick end labeling (TUNEL) staining was performed to detect the percentage of apoptotic cells in the jejuna and the ilea of piglets. Samples for TUNEL assay were prepared as described for intestinal morphology analysis. In summary, paraformaldehyde-fixed, paraffin-embedded full-thickness intestinal samples were cut into crosssections 5 μm thick, mounted on Superfrost Plus glass slides, deparaffinized, and rehydrated (xylenes, 100% ethanol, 90% ethanol, 70% ethanol, and double-distilled H_2_O). Following a PBS wash, the slices were incubated with the DNase-free protease K solution (20 μg/mL) for 30 min at 25 °C. To deactivate endogenous peroxides, the H_2_O_2_ solution (3% in PBS) was placed on the slides for 20 min at 25 °C. After that, the slices were washed in PBS three times and incubated with TUNEL reaction mixture (Beyotime Institute of Biotechnology, Haimen, China) for 60 min at 37 °C. The slices were rinsed again and incubated with horseradish peroxidase-conjugated streptavidin solution (Beyotime Institute of Biotechnology, China) for 30 min at 25 °C, and later with Di-amino-benzidine (DAB) chromogenic reagent (Beyotime Institute of Biotechnology, China) for 5 min at 25 °C. The slices were lightly counterstained with hematoxylin (Beyotime Institute of Biotechnology, China), dehydrated with ethanol, and mounted. The tissue sections were observed under the above mentioned Nikon ECLIPSE 80i light microscope (Nikon Corporation, Japan). The percentage of apoptotic cells (brown cells) was calculated by counting at least 200 cells in fifteen random villi per segment.

Ki-67 is a reliable marker for proliferation and mucosal crypt cell proliferation was determined using Ki-67 immunohistochemistry staining [32]. The samples were prepared as described above. The slides were treated with EDTA antigen retrieval for 25 min in a steamer. After quenching endogenous peroxidase and blocking nonspecific bindings, the slides were incubated with primary antibody (polyclonal rabbit anti-pig antibodies against Ki-67; Abcam, Cambridge, USA; 1:750) diluted in PBS for 20 min at 25 °C. Then, the sections were incubated with the biotinylated goat anti-rabbit secondary antibody for 20 min. After incubation with streptavidin peroxidase, the desired stain intensity was obtained with DAB by visualizing under the microscope. The slices were lightly counterstained with hematoxylin (Beyotime Institute of Biotechnology, China), dehydrated with ethanol, and mounted. Fifteen random crypts in each slice were analyzed. The total number of crypt cells and the number of Ki-67 positive cells (brown cells) in each crypt were counted to calculate the percentage of proliferative cells.

### Tissue homogenate

Approximately 2.0 g mucosal samples were minced and placed in ice-cold 154 mmol/L sterile sodium chloride solution (1:9, wt/vol), which were subsequently homogenized with an Ultra-Turrax homogenizer (Tekmar, Cincinnati, USA) at 13,500 r/m for 1 min. Then, the homogenate was centrifuged at 4,000 × *g* for 20 min at 4 °C, and the supernatant was analyzed rapidly. The protein concentrations in the homogenate were quantified using a bicinchoninic acid assay kit obtained from the Beyotime Institute of Biotechnology (China).

### Mucosal immune status assay

The levels of cytokines (tumor necrosis factor alpha (TNF-α), interferon gamma (IFN-γ), interleukin (IL)-1β, IL-4, IL-6, and IL-10) in the intestinal mucosa were determined using the ProcartaPlex™ multiplex immunoassay (Luminex, Austin, USA) kit according to the manufacturer’s instruction obtained from Affymetrix eBioscience (Santa Clara, USA). The concentrations of monocyte chemotactic protein 1 (MCP-1), mucin 2 (MUC2), and trefoil factor 3 (TFF3) were measured by ELISA using porcine-specific kits (CUSABIO Biotech, Wuhan, China). The detection ranges were 31.25 to 2,000 pg/mL for MCP-1, 7.8 to 500 ng/mL for MUC2, and 1.25 to 80 ng/mL for TFF3. The minimum detectable doses were < 7.81 pg/mL, < 1.95 ng/mL, and < 0.3125 ng/mL for MCP-1, MUC2, and TFF3, respectively. In addition, the inter- and intra-assay coefficients of variance were < 5% and < 10% for MCP-1, < 8% and < 10% for MUC2, and < 8% and < 10% for TFF3. Furthermore, myeloperoxidase (MPO) activity was measured using a colorimetric kit (Nanjing Jiancheng Institute of Bioengineering, Nanjing, China). All procedures were performed strictly following the manufacturers’ guidelines and the results were normalized against total protein concentration in each sample for inter-sample comparison.

### Quantification of intestinal microbiota

Bacterial DNA was extracted from the intestinal digesta samples using a TIANamp Stool DNA Kit (Tiangen, Beijing, China) according to the manufacturer’s instructions. The concentration and quality of DNA were analyzed using ultraviolet absorbance (NanoDrop Technologies, Wilmington, USA). The integrity of DNA was checked by electrophoresis on 1.5% agarose gels. The primers for the genus *Lactobacillus*, *Escherichia coli*, *Bacillus*, *Bifidobacterium*, and total bacteria are shown in Table 2 as reported previously [33–37]. After PCR amplification with Taq DNA polymerase kit (TaKaRa Biotechnology, Dalian, China) and electrophoresis on a 1.5% agarose gel, the PCR products were purified by a TIANgel Maxi Purification Kit (Tiangen, China) and cloned in *Escherichia coli* DH5α (Tiangen, China) using the pMD18-T vector system (TaKaRa Biotechnology, China). The extracted plasmids containing targeted fragments to be sequenced commercially, obtaining the positive plasmids (Invitrogen, Shanghai, China).

**Table 2:** Group-specific 16S–targeted primers and optimized conditions for real-time PCR

Name	Sequence, 5′ → 3’^a^	Annealing temperature, °C	Product size, bp	Reference
Total bacteria	ACTCCTACGGGAGGCAGCAG	60	200	[33]
	ATTACCGCGGCTGCTGG			
*Lactobacillus*	AGCAGTAGGGAATCTTCCA	55	341	[34]
	CACCGCTACACATGGAG			
*Escherichia coli*	CATGCCGCGTGTATGAAGAA	60	96	[35]
	CGGGTAACGTCAATGAGCAAA			
*Bacillus*	GCAACGAGCGCAACCCTTGA	60	92	[36]
	TCATCCCCACCTTCCTCCGGT			
*Bifidobacterium*	CTCCTGGAAACGGGTGG	55	550	[37]
	GGTGTTCTTCCCGATATCTACA			

^a^Shown as forward primer followed by reverse primer

The real-time absolute quantitative PCR reaction was applied to quantify the selected bacteria according to the method previously described [38]. Standard curves were generated with 10-fold serial dilutions of the respective positive plasmids. The concentration of the positive plasmids was plotted against the threshold cycle (Ct) value. The reaction was performed by SYBR Green fluorescence using the ABI StepOnePlus™ Real-Time PCR System (Applied Biosystems, Foster City, USA). The mixture (20 μL) of each reaction contained 2 μL DNA template, 0.8 μL of the primer pair, 10 μL of a freshly premixed SYBR® *Premix Ex Taq* (TaKaRa Biotechnology, China), 0.4 μL ROX reference dye (TaKaRa Biotechnology, China), and 6.8 μL double-distilled H_2_O. A control without template was included in all batches. The reaction protocol was composed of one cycle of denaturation at 95 °C for 30 s, forty cycles of denaturation at 95 °C for 5 s, followed by an annealing step according to the annealing temperature in Table 2 for 30 s, followed by a product melting curve to determine the specificity of amplification. Each sample was run in triplicate. The functions describing the relationship between Ct and *X* (log_10_ 16S rRNA gene copies/g contents) for the different assays were: Ct = − 0.257 *X* + 13.22; *R*^2^ = 0.9993 for total bacteria; Ct = − 0.301 *X *+ 13.37; *R*^2^ = 0.9992 for *Lactobacillus*; Ct = − 0.340 *X* + 14.41; *R*^2^ = 0.9996 for *Escherichia coli*; Ct = − 0.291 *X* + 12.8; *R*^2^ = 0.9992 for *Bacillus*; Ct = − 0.266 *X* + 12.32; *R*^2^ = 0.9990 for *Bifidobacterium*.

### Total RNA isolation and quantitative real-time PCR analysis

Total RNA was extracted from snap-frozen mucosal samples using the TRIzol Reagent (Invitrogen, Gaithersburg, USA). The concentration, quality, and integrity of RNA samples were performed as previously described [39]. After that, 1 μg of total RNA was reverse-transcribed into complementary DNA (cDNA) using a PrimeScripte RT Reagent Kit following the instruction provided by TaKaRa Biotechnology (China). Real-time PCR was performed using the ABI StepOnePlus™ Real-Time PCR System (Applied Biosystems, USA) with the One Step SYBR® PrimeScript™ RT-PCR Kit (TaKaRa Biotechnology, China) following the manufacturers’ instructions. Primer sequences of the target and reference genes (B-cell lymphoma/leukaemia 2 (*BCL-2*), myeloid cell leukemia 1 (*MCL-1*), B-cell lymphoma/leukaemia 2-associated X protein (*BAX*), phospholipid scramblase 3 (*PLSCR3*), glyceraldehyde phosphate dehydrogenase (*GAPDH*), and β-actin) are presented in Table 3. Verification of the specificity of each primer pair was performed using NCBI Blast (http://www.ncbi.nlm.nih.gov). The thermal cycling parameters were as follows: one cycle of denaturation at 95 °C for 30 s, forty cycles of denaturation at 95 °C for 5 s, followed by an annealing step at 60 °C for 30 s. To determine the specificity of amplification, a product melting curve analysis was carried out. In addition, the standard curve of each gene was run in triplicate for obtaining reliable amplification efficiency. The correlation coefficients of all standard curves were > 0.99 and the efficiency values were between 95 and 103%. The mRNA expression levels were calculated using the 2^-ΔΔCt^ method [40]. All the data were normalized to those of the reference gene *GAPDH*.

**Table 3:** Primer sequences used for real-time PCR assay

Name^a^	GenBank^b^	Sequence, 5′ → 3’^c^	Length, bp
*BCL-2*	XM_003121700.4	ATCAAGTGTTCCGCGTGACT	138
		GGGTACCAACAGCACCTCTC	
*MCL-1*	NM_214361.1	GCCTTTGTGGCCAAACACTT	129
		CCCATCCCAGCCTCTTTGTT	
*BAX*	XM_005664710.2	GAAACTCCTGGATCCGACGC	130
		TCTGGGGTTCTCCAGCTTCT	
*PLSCR3*	XM_005669207.2	TGTTCTAGGGGCTTCAGACG	158
		GTGTCCCGGAGGCTTAGTTC	
*GAPDH*	NM_001206359.1	CCAAGGAGTAAGAGCCCCTG	125
		AAGTCAGGAGATGCTCGGTG	
*β-actin*	XM_003124280.4	CTCCAGAGCGCAAGTACTCC	153
		AATGCAACTAACAGTCCGCC	

^a^*BAX* B-cell CLL/lymphoma 2-associated X protein, *β-actin* beta actin, *BCL-2* B-cell CLL/lymphoma 2, *GAPDH* glyceraldehyde phosphate dehydrogenase, *MCL-1* myeloid cell leukemia 1, *PLSCR3* phospholipid scramblase 3

^b^GenBank Accession Number

^c^Shown as forward primer followed by reverse primer

### Statistical analysis

Data were tested for normality (Shapiro-Wilk test) and homogeneity of variances (Levene’s test) prior to statistical analysis. Data that were heterogeneous or not normally distributed were analyzed using non-parametric Kruskal-Wallis test, and pairwise differences in rank sums were evaluated using selected comparisons tests. All normal data were tested for statistical significance using one-way ANOVA and Tukey’s post hoc test for pairwise comparisons. Data were analyzed by using SPSS statistical software (ver. 22.0 for Windows, SPSS Inc., Chicago, USA). The *P* values less than 0.05 were considered as statistically significant. Results are presented as means with standard errors of the mean (SEM).

## Results

### Growth performance

During the first 4 wk after weaning, ADG and ADFI were significantly lower in the IUGR-CON piglets than in the NBW-CON piglets (*P* < 0.05; Table 4). Supplementation with BA effectively improved the growth performance of the IUGR-BA group, as indicated by their increases in ADG and FE (*P* < 0.05). However, the BA-supplemented diet did not alter the ADFI of the piglets (*P* > 0.05).

**Table 4:** Effects of *Bacillus amyloliquefaciens* on growth performance of weanling piglets with intra-uterine growth retardation

Items^a,b^	NBW-CON (NC group)	IUGR-CON (IC group)	IUGR-BA (IB group)	SEM	Contrast	
					NC vs. IC	IC vs. IB
ADG^b^, g/d	362.74	255.89	301.85	12.31	< 0.001	0.033
ADFI^b^, g/d	577.23	429.02	470.02	18.44	< 0.001	0.320
FE^b^, g/g	0.63	0.60	0.64	0.01	0.115	0.026

^a^*ADFI* average daily feed intake, *ADG* average daily weight gain, *FE* feed efficiency, *IUGR-BA* piglets with intrauterine growth retardation fed the *Bacillus amyloliquefaciens*-supplemented diet, *IUGR-CON* piglets with intrauterine growth retardation fed the control diet, *NBW-CON* piglets with normal birth weight fed the control diet

^b^One-way ANOVA test. A value of *P* < 0.05 was considered as statistically significant

### Intestinal morphology, cell proliferation, and apoptosis

Morphological results indicated that IUGR reduced the VH and the ratio of VH to CD in the jejunum and in the ileum (*P* < 0.05), whereas the CD was not altered (*P* > 0.05; Table 5). In contrast, the jejunal VH:CD ratio and ileal VH were greater in the IUGR-BA piglets than in the non-treated IUGR piglets (*P* < 0.05).

**Table 5:** Effects of *Bacillus amyloliquefaciens* on intestinal morphology of weanling piglets with intra-uterine growth retardation

Items^a^		NBW-CON (NC group)	IUGR-CON (IC group)	IUGR-BA (IB group)	SEM	Contrast	
						NC vs. IC	IC vs. IB
Jejunum	VH^c^, μm	498.79	389.69	443.27	14.48	0.004	0.433
	CD^b^, μm	198.40	210.17	197.76	5.81	0.709	0.683
	VH:CD ratio^b^, μm/μm	2.54	1.88	2.24	0.08	< 0.001	0.040
Ileum	VH^b^, μm	414.52	352.21	400.62	9.12	0.005	0.029
	CD^c^, μm	177.82	185.08	175.72	3.87	NS	NS
	VH:CD ratio^b^, μm/μm	2.34	1.92	2.30	0.07	0.035	0.063

^a^*CD* crypt depth, *IUGR-BA* piglets with intrauterine growth retardation fed the *Bacillus amyloliquefaciens*-supplemented diet, *IUGR-CON* piglets with intrauterine growth retardation fed the control diet, *NBW-CON* piglets with normal birth weight fed the control diet, *NS* nonsignificant values after Kruskal–Wallis comparison test, *VH* villus height, *VH:CD ratio* the ratio of villus height to crypt depth

^b^One-way ANOVA test. A value of *P* < 0.05 was considered as statistically significant

^c^Non-parametric Kruskal-Wallis test. A value of *P* < 0.05 was considered as statistically significant

**Fig. 2:**
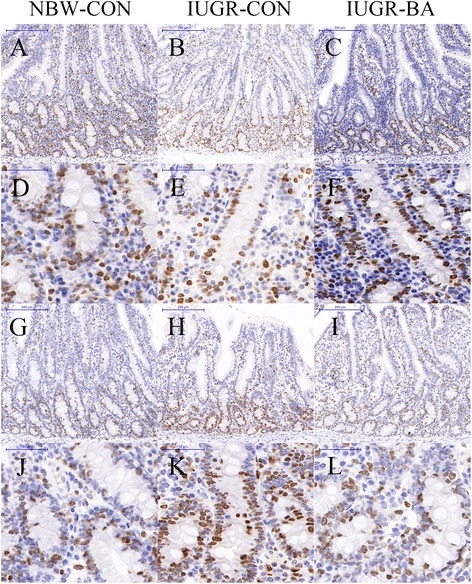
Representative micrographs of Ki-67 staining carried out on paraformaldehyde-fixed sections from the jejunum [(**a**–**c**), 100 × magnification; (**d**–**f**), 400 × magnification] and ileum [(**g**–**i**), 100 × magnification; (**j**–**l**), 400 × magnification] of weanling piglets. *IUGR-BA* piglets with intrauterine growth retardation fed the *Bacillus amyloliquefaciens*-supplemented diet, *IUGR-CON* piglets with intrauterine growth retardation fed the control diet, *NBW-CON* piglets with normal birth weight fed the control diet

**Fig. 3:**
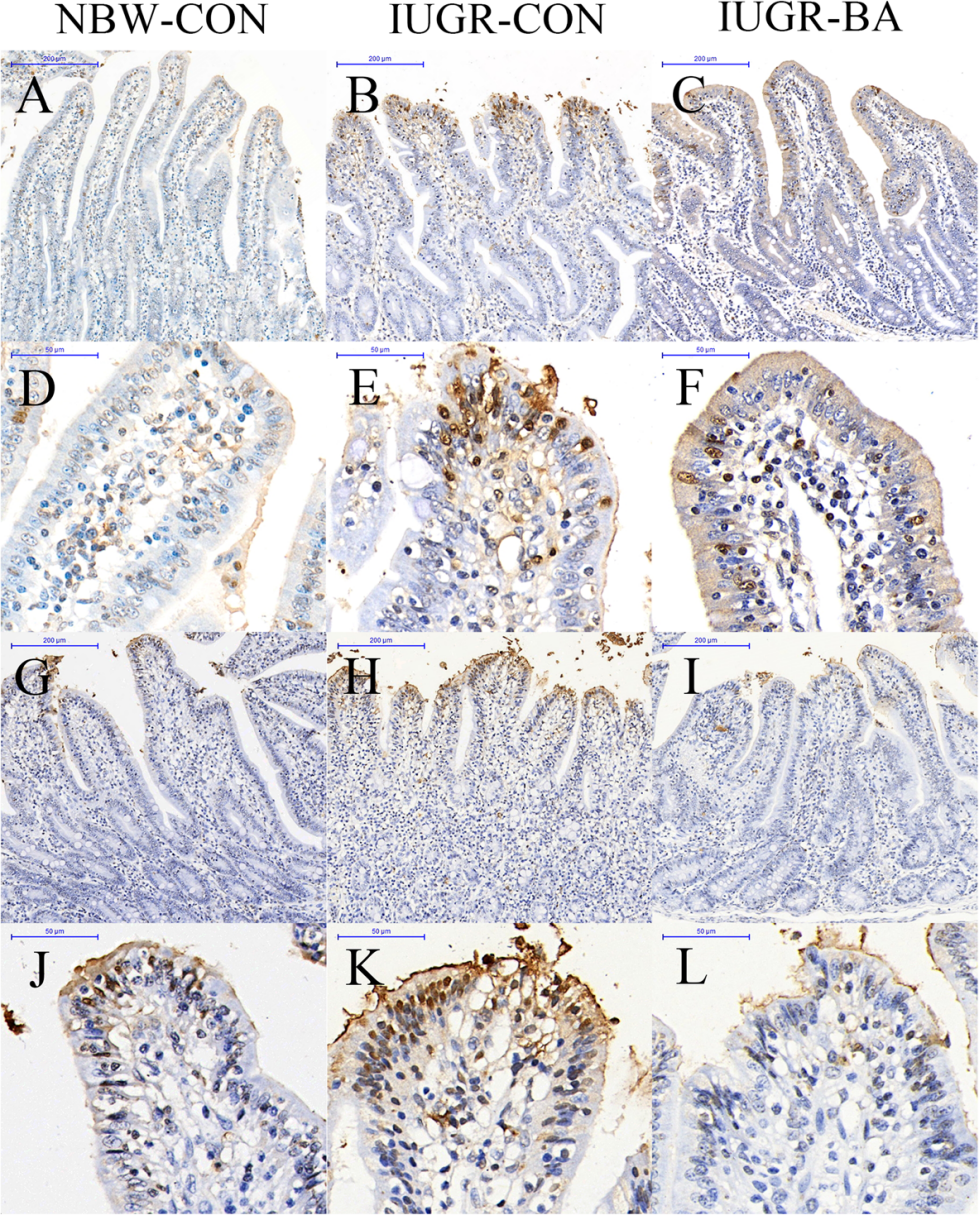
Representative micrographs of TUNEL staining carried out on paraformaldehyde-fixed sections from the jejunum [(**a**–**c**), 100 × magnification; (**d**–**f**), 400 × magnification] and ileum [(**g**–**i**), 100 × magnification; (**j**–**l**), 400 × magnification] of weanling piglets. *IUGR-BA* piglets with intrauterine growth retardation fed the *Bacillus amyloliquefaciens*-supplemented diet, *IUGR-CON* piglets with intrauterine growth retardation fed the control diet, *NBW-CON* piglets with normal birth weight fed the control diet

Compared with the NBW-CON piglets, IUGR induced a significantly increased proliferation of ileal crypt cells (Fig. 2), but there was no effect in the jejunum (*P* < 0.05; Table 6). The numbers of apoptotic cells (Fig. 3) were greater in the jejuna and the ilea of IUGR-CON group than of the NBW-CON group (*P* < 0.05). Conversely, apoptosis rates were significantly decreased in the small intestines of the IUGR piglets fed the BA-supplemented diet compared with those fed the control diet (*P* < 0.05). In addition, the IUGR-BA group showed a lower proportion than the IUGR-CON group of crypt proliferative cells in their ilea (*P* < 0.05).

**Table 6:** Effects of *Bacillus amyloliquefaciens* on intestinal cell proliferation and apoptosis of weanling piglets with intra-uterine growth retardation

Items^a^		NBW-CON (NC group)	IUGR-CON (IC group)	IUGR-BA (IB group)	SEM	Contrast	
						NC vs. IC	IC vs. IB
Jejunum	Ki-67 positive cell percentage per crypt^b^, %	42.28	46.25	41.10	1.21	0.366	0.198
	TUNEL positive cell percentage per 100 cells of villus^b^, %	7.82	12.46	9.88	0.58	0.001	0.038
Ileum	Ki-67 positive cell percentage per crypt^b^, %	39.79	50.55	39.02	1.93	0.030	0.020
	TUNEL positive cell percentage per 100 cells of villus^b^, %	8.06	13.93	6.93	0.91	0.002	< 0.001

^a^*IUGR-BA* piglets with intrauterine growth retardation fed the *Bacillus amyloliquefaciens*-supplemented diet, *IUGR-CON* piglets with intrauterine growth retardation fed the control diet, *NBW-CON* piglets with normal birth weight fed the control diet

^b^One-way ANOVA test. A value of *P* < 0.05 was considered as statistically significant

The expression levels of genes related to apoptosis are summarized in Table 7. IUGR was found to decrease *BCL-2* mRNA abundance in the jejuna and the ilea of the IUGR-CON piglets compared to the NBW-CON piglets (*P* < 0.05). However, the mRNA levels of intestinal *MCL-1*, *BAX*, and *PLSCR3* were not affected by IUGR (*P* > 0.05). In addition, BA had no effect on the expression levels of BCL-2, MCL-1, BAX, or PLSCR3 (*P* > 0.05).

**Table 7:** Effects of *Bacillus amyloliquefaciens* on apoptosis-associated gene expression of weanling piglets with intra-uterine growth retardation

Items^a^		NBW-CON (NC group)	IUGR-CON (IC group)	IUGR-BA (IB group)	SEM	Contrast	
						NC vs. IC	IC vs. IB
Jejunum	*BCL-2* ^c^	1.00	0.53	0.80	0.08	0.033	0.092
	*MCL-1* ^b^	1.00	0.66	0.85	0.07	0.081	0.402
	*BAX* ^b^	1.00	1.61	1.27	0.13	0.123	0.491
	*PLSCR3* ^b^	1.00	1.08	1.03	0.14	0.969	0.988
Ileum	*BCL-2* ^b^	1.00	0.53	0.89	0.08	0.017	0.067
	*MCL-1* ^b^	1.00	0.81	1.01	0.08	0.626	0.606
	*BAX* ^c^	1.00	1.64	1.08	0.11	NS	NS
	*PLSCR3* ^b^	1.00	0.94	0.86	0.07	0.952	0.890

^a^*BAX* B-cell lymphoma/leukaemia 2-associated X protein, *BCL-2* B-cell CLL/lymphoma 2, *IUGR-BA* piglets with intrauterine growth retardation fed the *Bacillus amyloliquefaciens*-supplemented diet, *IUGR-CON* piglets with intrauterine growth retardation fed the control diet, *NBW-CON* piglets with normal birth weight fed the control diet, *NS* nonsignificant values after Kruskal–Wallis comparison test, *MCL-1* myeloid cell leukemia 1, *PLSCR3* phospholipid scramblase 3

^b^One-way ANOVA test. A value of *P* < 0.05 was considered as statistically significant

^c^Non-parametric Kruskal-Wallis test. A value of *P* < 0.05 was considered as statistically significant

### Intestinal immune status

MCP-1 concentration and MPO activity were higher in the jejuna and the ilea of the IUGR-CON piglets than of the NBW-CON piglets (*P* < 0.05; Table 8). A similar effect induced by IUGR was observed for TNF-α and IL-1β levels in the jejuna of the control piglets (*P* < 0.05). IUGR significantly decreased the ileal IL-10 content of the control piglets compared with the NBW-CON piglets (*P* < 0.05). However, feeding the IUGR piglets with the BA-supplemented diet effectively increased IL-10 content but decreased TNF-α level in both the jejunum and ileum compared with the control diet (*P* < 0.05). In the ileum, BA supplementation decreased the activity of MPO in the IUGR-BA group compared with the IUGR-CON group (*P* < 0.05). Neither IUGR nor BA supplementation affected the IFN-γ, IL-4, or IL-6 levels in the small intestine (*P* > 0.05).

**Table 8:** Effects of *Bacillus amyloliquefaciens* on intestinal immune status of weanling piglets with intra-uterine growth retardation

Items^a^		NBW-CON (NC group)	IUGR-CON (IC group)	IUGR-BA (IB group)	SEM	Contrast	
						NC vs. IC	IC vs. IB
Jejunum	TNF-α^b^, pg/mg protein	0.90	1.61	0.84	0.13	0.038	0.024
	IFN-γ^b^, pg/mg protein	1.52	2.13	1.44	0.13	0.093	0.054
	IL-1β^b^, pg/mg protein	4.37	8.56	5.49	0.64	0.011	0.062
	IL-4^b^, pg/mg protein	1.26	1.27	1.54	0.08	0.998	0.406
	IL-6^b^, pg/mg protein	1.71	3.10	2.34	0.29	0.120	0.495
	IL-10^b^, pg/mg protein	19.09	14.40	22.22	1.30	0.227	0.029
	MCP-1^b^, pg/mg protein	5.30	7.30	5.88	0.34	0.031	0.140
	MPO^c^, IU/g protein	25.24	61.00	29.68	5.25	0.005	0.052
Ileum	TNF-α^b^, pg/mg protein	1.20	1.68	1.00	0.10	0.082	0.012
	IFN-γ^b^, pg/mg protein	1.93	2.24	1.57	0.15	0.632	0.148
	IL-1β^c^, pg/mg protein	6.66	13.78	7.06	1.17	0.092	0.080
	IL-4^b^, pg/mg protein	0.79	0.80	1.06	0.06	0.999	0.211
	IL-6^b^, pg/mg protein	1.80	1.84	2.03	0.15	0.994	0.872
	IL-10^c^, pg/mg protein	22.38	12.30	25.76	1.79	0.028	0.004
	MCP-1^b^, pg/mg protein	5.37	8.67	7.17	0.54	0.025	0.394
	MPO^b^, IU/g protein	38.41	73.12	49.48	4.59	0.001	0.020

^a^*IFN-γ* interferon gamma, *IL-1β* interleukin 1 beta, *IL-4* interleukin 4; *IL-6* interleukin 6, *IL-10* interleukin 10, *IUGR-BA* piglets with intrauterine growth retardation fed the *Bacillus amyloliquefaciens*-supplemented diet, *IUGR-CON* piglets with intrauterine growth retardation fed the control diet, *MCP-1* monocyte chemotactic protein 1, *MPO* myeloperoxidase, *NBW-CON* piglets with normal birth weight fed the control diet, *TNF-α* tumor necrosis factor alpha

^b^One-way ANOVA test. A value of *P* < 0.05 was considered as statistically significant

^c^Non-parametric Kruskal-Wallis test. A value of *P* < 0.05 was considered as statistically significant

**Fig. 4:**
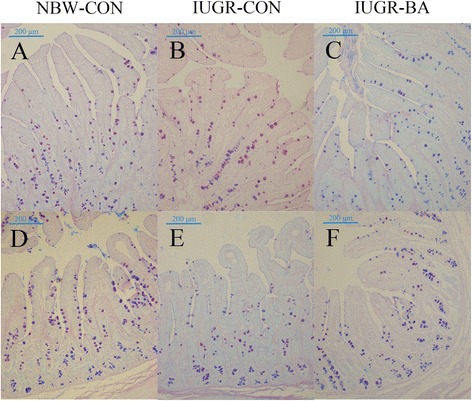
Representative micrographs of goblet cell staining carried out on paraformaldehyde-fixed sections from the jejunum [(**a**–**c**), 100 × magnification] and ileum [(**d**–**f**), 100 × magnification] of weanling piglets. *IUGR-BA* piglets with intrauterine growth retardation fed the *Bacillus amyloliquefaciens*-supplemented diet, *IUGR-CON* piglets with intrauterine growth retardation fed the control diet, *NBW-CON* piglets with normal birth weight fed the control diet

As shown in Table 9, IUGR decreased TFF3 content but had no effect on goblet cell numbers (Fig. 4) or MUC2 content in the jejuna of the IUGR-CON group, when compared with the NBW-CON group (*P* < 0.05). In the ileum, goblet cell density and MUC2 and TFF3 concentrations were greatly reduced in the IUGR-CON piglets compared to the NBW-CON group (*P* < 0.05). However, the adverse effects that IUGR exerted in ileal goblet cell density and MUC2 and TFF3 productions were attenuated by BA supplementation (*P* < 0.05). In addition, feeding the IUGR piglets with the BA diet did not alter these parameters in the jejunum (*P* > 0.05).

**Table 9:** Effects of *Bacillus amyloliquefaciens* on intestinal goblet cell density and mucin 2 and trefoil factor 3 contents of weanling piglets with intra-uterine growth retardation

Items^a^		NBW-CON (NC group)	IUGR-CON (IC group)	IUGR-BA (IB group)	SEM	Contrast	
						NC vs. IC	IC vs. IB
Jejunum	Goblet cell density^b^, n/100 μm of villus height	2.89	2.69	2.72	0.07	0.540	0.982
	MUC2^c^, ng/mg protein	12.52	8.04	8.92	0.95	NS	NS
	TFF3^b^, ng/mg protein	107.69	83.22	93.88	3.72	0.012	0.340
Ileum	Goblet cell density^b^, n/100 μm of villus height	4.02	3.16	3.84	0.13	0.009	0.039
	MUC2^b^, ng/mg protein	17.75	6.99	13.89	1.36	< 0.001	0.016
	TFF3^b^, ng/mg protein	168.07	103.83	151.02	9.17	0.004	0.032

^a^*IUGR-BA* piglets with intrauterine growth retardation fed the *Bacillus amyloliquefaciens*-supplemented diet, *IUGR-CON* piglets with intrauterine growth retardation fed the control diet, *MUC2* mucin 2, *NBW-CON* piglets with normal birth weight fed the control diet, *NS* nonsignificant values after Kruskal–Wallis comparison test, *TFF3* trefoil factor 3

^b^One-way ANOVA test. A value of *P* < 0.05 was considered as statistically significant

^c^Non-parametric Kruskal-Wallis test. A value of *P* < 0.05 was considered as statistically significant

### Microbiota populations

The IUGR-CON piglets showed increased *Escherichia coli* but decreased *Bifidobacterium* abundance in the ileal digesta compared with the NBW-CON piglets (*P* < 0.05; Table 10). Conversely, the numbers of *Escherichia coli* were significantly lower in the jejunal contents of the IUGR-BA piglets than those of the IUGR-CON piglets (*P* < 0.05). Supplementation of BA increased the populations of *Lactobacillus* and *Bifidobacterium* in the ileal digesta of the IUGR-BA group in comparison with the IUGR-CON group (*P* < 0.05). No differences were found among the groups in respect of abundance of total bacteria or *Bacillus* (*P* > 0.05).

**Table 10:** Effects of *Bacillus amyloliquefaciens* on intestinal microbiota abundance of weanling piglets with intra-uterine growth retardation

Items^a^		NBW-CON (NC group)	IUGR-CON (IC group)	IUGR-BA (IB group)	SEM	Contrast	
						NC vs. IC	IC vs. IB
Jejunum	Total bacteria^b^, log_10_ copies/g	9.52	9.65	9.50	0.11	0.888	0.857
	*Lactobacillus*^b^, log_10_ copies/g	6.75	6.61	7.09	0.17	0.942	0.502
	*Escherichia coli*^c^, log_10_ copies/g	6.20	6.87	6.00	0.14	0.223	0.028
	*Bacillus*^b^, log_10_ copies/g	4.38	4.39	4.69	0.11	1.000	0.507
	*Bifidobacterium*^b^, log_10_ copies/g	2.90	2.82	3.12	0.09	0.931	0.398
Ileum	Total bacteria^c^, log_10_ copies/g	10.12	10.26	10.20	0.09	NS	NS
	*Lactobacillus*^b^, log_10_ copies/g	7.79	7.02	8.03	0.17	0.105	0.030
	*Escherichia coli*^c^, log_10_ copies/g	6.12	7.20	6.39	0.16	0.033	0.198
	*Bacillus*^c^, log_10_ copies/g	4.85	4.63	5.37	0.15	NS	NS
	*Bifidobacterium*^b^, log_10_ copies/g	3.65	3.00	3.76	0.11	0.019	0.007

^a^*IUGR-BA* piglets with intrauterine growth retardation fed the *Bacillus amyloliquefaciens*-supplemented diet, *IUGR-CON* piglets with intrauterine growth retardation fed the control diet, *NBW-CON* piglets with normal birth weight fed the control diet, *NS* nonsignificant values after Kruskal–Wallis comparison test

^b^One-way ANOVA test. A value of *P* < 0.05 was considered as statistically significant

^c^Non-parametric Kruskal-Wallis test. A value of *P* < 0.05 was considered as statistically significant

## Discussion

It has been reported that IUGR affects the development of the gastrointestinal tract and impairs the morphology of the small-intestinal mucosa in humans and in several animal models. The key role of the intestine in processing dietary molecules into available nutrients for the organism makes this a matter of great importance [41, 42]. The present study corroborates previous findings and suggests that the lower villus sizes were probably related to the disturbance in the balance of cell proliferation and apoptosis. Excessive apoptosis of villus cells can cause shortening of the villi [43], and the TUNEL staining showed higher rates of apoptotic cells in both the jejuna and the ilea of the IUGR piglets. Additionally, the intestinal expression pattern of apoptosis-related genes of the IUGR-CON piglets differed from that of their heavier counterparts. Consistent with the increased proportion of cell apoptosis, a decreased abundance of *BCL-2* mRNA was noted in the IUGR-CON piglets, which may reflect compromised anti-apoptotic function of BCL-2 in the small intestine. Similar results have been seen in rodent models of IUGR, where levels of *BCL-2* mRNA were significantly down-regulated in the brain, kidneys, and ileum [3, 44, 45]. BCL-2 is an anti-apoptotic protein that attenuates the release of cytochrome c from mitochondria and counteracts the effects of BAX. Low levels of BCL-2 may be responsible for the higher apoptotic index in the small intestines of the IUGR piglets. Moreover, it is noteworthy that IUGR induced a greater proportion of crypt proliferative cells in the ilea of the IUGR-CON piglets, which may imply a compensatory process in response to the excessive apoptosis in the villus.

Impairment of intestinal structure resulting from IUGR could induce a vicious cycle of bacterial invasion, immune activation, and uncontrolled inflammation. Indeed, IUGR is known to predispose neonates to intestinal inflammatory diseases [46]. The mechanism by which this occurs involves an imbalance of pro- and anti-inflammatory cytokines. The present study observed higher levels of pro-inflammatory cytokines TNF-α and IL-1β in the IUGR-CON group, but a lower content of anti-inflammatory cytokine IL-10. Contrary to these results, Han et al. [47] found that IUGR decreased the mRNA abundance of IL-1β in the ilea of IUGR neonates. This discrepancy between the studies is likely due to the stage of growth. Weaning is associated with a transient inflammation of the gut, in parallel with an increased plasma IL-1β concentration and with gene expression in the intestines of piglets [48, 49]. However, the weaning-associated intestinal inflammation usually becomes more serious and long-lasting with light piglets at birth, most likely owing to a postnatal delay in the maturation of the immune defense system.

Goblet cells can synthesize and secrete bioactive molecules that are components of mucus, such as mucins and trefoil factors [50]. The mucus layer coating the intestinal epithelium forms a physical barrier that protects against endogenous and exogenous irritants and microbial attachment and invasion [51]. So far, a number of mucins have been identified; among them, MUC2 is the predominant secretory mucin in the small intestine [52]. In this study, levels of MUC2 and TFF3 were lower in the intestines of the IUGR-CON piglets, particularly in the ilea, where the number of goblet cells was dramatically reduced. Of the possible mechanisms by which IUGR inhibits intestinal goblet cell density, one is overproduction of pro-inflammatory cytokines in response to potential inflammatory stimuli, which can lead to depletion of goblet cells [53]. Another possible explanation is the longer physical adaptation for weaning stress in the IUGR piglets, which would delay the process of the intestine regaining normal goblet cell function. Normally, goblet cell numbers in the villi decrease during the weaning process and begin to increase again from 3 to 15 days post-weaning [54]. However, IUGR has been found to reduce the numbers of colonic goblet cells in rats post-weaning (and even in young adult rats), and it is known to be accompanied by a reduction of MUC2 at both the mRNA and protein levels [55]. In fact, emerging evidence indicates that a delay in the development of the mucus barrier—and, in particular, a lower capacity of goblet cells for producing mucus as a response to infection—may explain why IUGR neonates are more susceptible to intestinal diseases [56, 57].

Further, the IUGR-CON piglets in the present study exhibited an elevated activity of ileal MPO, an enzyme which participates in tissue injury in a large number of inflammatory conditions by producing cytotoxic oxidants [58]. This increase may be a sign of inflammatory status. Moreover, an increased level of chemokine MCP-1, a potent chemoattractant capable of promoting monocyte recruitment into an inflammatory site, was observed in the intestines of the IUGR piglets. This would enable recruited cells to produce more pro-inflammatory mediators, thereby potentiating inflammation. If an inflammation cannot be controlled promptly, it will have adverse consequences for the growth performance of animals, mainly by diverting available nutrients away from growth processes and toward immune-related processes [59].

Probiotic bacteria can regulate lymphocyte cytokine production and inflammatory responses [60]. Hairul Islam et al. [61] reported that BA could alleviate increased concentrations of pro-inflammatory mediators, such as TNF-α and IL-1β in a mouse model of colitis induced by dextran sulfate. The results of the present study further corroborate the ability of BA to temper IUGR-induced excessive secretion of TNF-α in the small intestine, which may be due to the simultaneous increase in IL-10 content after administration of BA. IL-10 has a potent anti-inflammatory effect in decreasing the overproduction of pro-inflammatory cytokines under immunological stress [62]. Thus, supplementation with BA may have helped to alleviate intestinal inflammatory responses in the IUGR piglets (probably by improving the balance of pro- and anti-inflammatory cytokines), and this beneficial effect was also supported by the decrease in MPO activity after BA-supplemented feeding.

The underlying mechanisms through which BA regulates the inflammatory status may relate to the release of surfactin. Surfactin is a bacterium-derived molecule that can be produced by several *Bacillus* species including BA. Eun et al. [63] revealed that surfactin is able to reduce the release of inflammation-related mediators, first by blocking the phosphorylation of inhibitor of nuclear factor kappa-B kinase, and then by nuclear translocation of nuclear factor-kappa B (NF-κB). In addition to inhibiting NF-κB activation, surfactin was found to suppress the signaling of signal transducer and activator of transcription (STAT)-1, which could facilitate the production of many pro-inflammatory molecules [64]. Meanwhile, Park et al. [64] found that surfactin increased the phosphorylation of STAT-3, a component of the homeostatic mechanism inducing anti-inflammatory gene transcription, such as IL-10 [65]. This observation may provide a mechanistic explanation for the increased IL-10 secretion observed in the intestines of IUGR-BA piglets. Similarly, a recent study reported that surfactin produced from a BA culture increased the secretion of IL-10 in the splenocytes of non-obese diabetic mice [66]. Thus, BA may have therapeutic potential to treat intestinal inflammation caused by IUGR.

The above-mentioned findings are also supported by morphologic observation. The changes in small-intestinal morphology observed here for the IUGR-BA piglets, and in particular the increases in VH and VH:CD ratio, may indicate an improvement of intestinal health status. These observations are consistent with the findings of Cai et al. [67] in their study of weanling piglets receiving a BA-based direct-fed-microbial. Moreover, a recent study demonstrated a higher VH and a lower CD in the ilea of IUGR suckling piglets given diets that included *Bacillus subtilis* [68]. Subsequent TUNEL analysis revealed that supplementation with BA attenuated the increased apoptotic index in both the jejunum and ileum induced by IUGR. This action of BA in limiting excessive inflammation may provide a mechanistic explanation for the improved intestinal structure and cellular homeostasis. Other studies have provided significant evidence that probiotics and probiotic-derived soluble factor prevent epithelial apoptosis and the disruption of barrier function induced by pro-inflammatory mediators [27, 64, 68, 69]. Furthermore, the present findings indicate that supplementation with BA strongly promoted the secretions of MUC2 and TFF3 in the ilea of the IUGR-BA piglets, which may be related to the simultaneous increase in goblet cell numbers. Similar results have also been achieved by the addition of various probiotics, such as VSL#3 (a probiotic mixture containing four species of *Lactobacilli* and three species of *Bifidobacteria*) [70], *Lactobacillus rhamnosus* GG [71], *Bacillus subtilis* [72, 73], and BA [61, 74]. It is of particular interest that TFF3 has been shown to play an essential role in the maintenance and repair of the intestinal mucosa [75]. These effects may therefore indicate that BA has the potential to repair the impaired intestinal structure of newly weaned piglets with IUGR.

The present study revealed that the IUGR-CON piglets had less *Bifidobacterium* and more *Escherichia coli* in their ileal digesta than their non-IUGR littermates. This is in line with the findings of a previous study that found a lower amount of *Bifidobacterium* in the cecocolonic content of IUGR rats at several time points from birth to sexual maturation [15]. One possible explanation for perturbations in the normal pattern of microbiota in the IUGR-CON piglets may be associated with decreases in goblet cell numbers and MUC2 and TFF3 contents, as mentioned above. Changes in goblet cell function and in the chemical composition of intestinal mucus have been detected in response to a broad range of luminal insults, including alterations of the normal microbiota [76]. However, the IUGR-induced decrease in the *Bifidobacterium* population was counteracted by the administration of BA. A higher population of *Lactobacillus* was also noted in the IUGR-BA group. In addition to stimulating goblet cell function, BA serves as an aerobic bacterial species that can consume oxygen rapidly and then provide a favorable environment for the colonization of *Lactobacillus* and *Bifidobacterium.* Furthermore, BA has the ability to stimulate the secretion of lactic acid and thereby induces a drop in intestinal pH, which is beneficial to the growth of *Lactobacillus* [77, 78].

Additionally, the inhibition of BA against *Escherichia coli* was observed in the jejunal contents of the IUGR-BA piglets in the current study. We previously found that BA is highly effective against potential pathogenic bacteria such as *Escherichia coli*, *Bacillus cereus*, and *Salmonella typhimurium* [79, 80], which indicates the potential of BA to inhibit pathogens in the gut. In poultry, BA has also been found to inhibit the growth of the pathogenic bacteria *Clostridium perfringens* and *Escherichia coli*, and to improve the feed conversion rate in poultry [28, 81]. BA can secrete bacteriocins including subtilin and barnase, which suppress the growth of pathogens directly, and antimicrobial peptides produced by BA have been verified as bactericidal agents [82, 83]. Furthermore, increased numbers of *Lactobacillus* and *Bifidobacterium* may help to inhibit the growth of *Escherichia coli*, possibly by a phenomenon known as competitive exclusion [84]. Taken together, these effects indicate that BA may be beneficial in stabilizing the intestinal ecosystem of IUGR piglets by replenishing suppressed health-promoting bacteria and inhibiting the growth of potential pathogenic microbiota.

## Conclusions

The results obtained in the present study provide evidence that the inclusion of BA in diets for the IUGR piglets contributes in a number of ways to their improved growth performance during the first 4 wk post-weaning, including improvements in intestinal morphology, a decrease in inflammatory response, and better regulation of microbiota. The findings of this study may therefore help in the development of novel nutrition strategies for IUGR infants to prevent intestinal diseases in early life.

## Additional file

Additional file 1: Availability of data and materials. (XLSX 32 kb)

## Abbreviations

ADFI: Average daily feed intake
ADG: Average daily gain
BA: *Bacillus amyloliquefaciens*
BAX: B-cell lymphoma/leukaemia 2-associated X protein
BCL-2: B-cell lymphoma/leukaemia 2
BW: Birth weight
CD: Crypt depth
cDNA: complementary DNA
Ct: Threshold cycle
DAB: Di-amino-benzidine
FE: Feed efficiency
GAPDH: Glyceraldehyde phosphate dehydrogenase
IFN-γ: Interferon gamma
IL: Interleukin
IUGR: Intra-uterine growth retardation
MCL-1: Myeloid cell leukemia 1
MCP-1: Monocyte chemotactic protein 1
MPO: Myeloperoxidase
MUC2: Mucin 2
NBW: Normal body weight
NF-κB: Nuclear factor-kappa B
NS: Not significant
PLSCR3: Phospholipid scramblase 3
STAT: Signal transducer and activator of transcription
TFF3: Trefoil factor 3
TNF-α: Tumor necrosis factor alpha
TUNEL: Terminal deoxynucleotidyl transferase-mediated deoxyuridine triphosphate nick end labeling
VH: Villus height
